# Kidney disease models: tools to identify mechanisms and potential therapeutic targets

**DOI:** 10.24272/j.issn.2095-8137.2017.055

**Published:** 2018-03-07

**Authors:** Yin-Wu Bao, Yuan Yuan, Jiang-Hua Chen, Wei-Qiang Lin

**Affiliations:** 1 Kidney Disease Center, First Affiliated Hospital, School of Medicine, Zhejiang University, Hangzhou Zhejiang 310058, China; 2 Institute of Translational Medicine, School of Medicine, Zhejiang University, Hangzhou Zhejiang 310058, China

**Keywords:** Acute kidney injury, Chronic kidney disease, Mouse models, Transgenic mice

## Abstract

Acute kidney injury (AKI) and chronic kidney disease (CKD) are worldwide public health problems affecting millions of people and have rapidly increased in prevalence in recent years. Due to the multiple causes of renal failure, many animal models have been developed to advance our understanding of human nephropathy. Among these experimental models, rodents have been extensively used to enable mechanistic understanding of kidney disease induction and progression, as well as to identify potential targets for therapy. In this review, we discuss AKI models induced by surgical operation and drugs or toxins, as well as a variety of CKD models (mainly genetically modified mouse models). Results from recent and ongoing clinical trials and conceptual advances derived from animal models are also explored.

## INTRODUCTION

Foundation items: This study was supported by the General Program of the National Natural Science Foundation of China (51309220, 31470776) and QianJiang Talent Plan to W.Q. Lin

Acute kidney injury (AKI) and chronic kidney disease (CKD) are linked to high morbidity and mortality. AKI is regarded as a rapid and reversible decline in renal function and is associated with accelerated CKD (Siew & Davenport, 2015). The ability to diagnose AKI has progressed significantly. Recent consensus diagnostic criteria include an increase in serum creatinine ≥0.3 mg/dL (≥26.5 µmol/L) within 48 h; an increase in serum creatinine to ≥1.5 times baseline; or urine volume <0.5 mL/kg/h for 6 h (Khwaja, 2012). Many risk factors such as drugs/toxins, sepsis, and ischemia-reperfusion (IR) commonly result in AKI and lead to reduced glomerular filtration rate (GFR) as well as acute tubular cell death (Sanz et al., 2013). CKD is a significant medical problem globally, with a rapid increase in incidence due to the rise in hypertension and diabetes (Tomino, 2014). CKD is usually diagnosed by the presence of albuminuria or estimated GFR from serum creatinine <60 mL/min/1.73 m^2^ (Andrassy, 2013).

**Figure 1:**
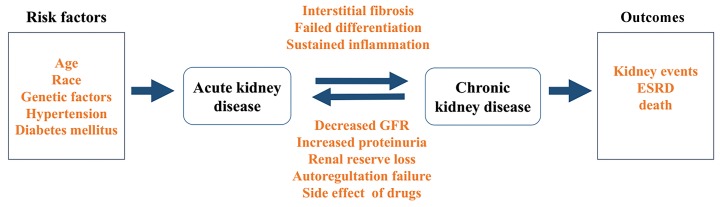
Relationship between acute kidney injury (AKI) and chronic kidney disease (CKD)

There is increasing recognition that AKI and CKD are closely linked and are therefore regarded as an integrated clinical syndrome (Chawla & Kimmel, 2012) (Figure 1). Key biological processes such as cell death, cell proliferation, inflammation, and fibrosis, as well as common biomarkers, are detected in both kinds of nephropathy (Andreucci et al., 2017; Endre et al., 2011). Generally, tubular cell death, which includes necrosis, apoptosis, or necroptosis, is the main histological feature in early stage AKI, whereas fibrosis tends to occur under CKD. An increasing number of studies have shown that AKI is a major risk factor that can accelerate CKD progression (Pannu, 2013). Clinical observations have also found a strong relationship between AKI and CKD. Compared to patients with no history of AKI or CKD, AKI patients are more likely to develop new CKD or end-stage renal disease (ESRD) (Chawla et al., 2014; Chawla & Kimmel, 2012). Conversely, CKD also plays an important role in AKI. Patients with CKD may suffer higher risk of transient decreases in renal function consistent with AKI (Chawla et al., 2014). The underlying mechanism that results in acute renal dysfunction may involve decreased GFR, increased proteinuria, renal auto-regulation failure, and drug side effects (Hsu & Hsu, 2016).

AKI and CKD show some common risk factors such as age, race, and hypertension. Persistent AKI can induce interstitial fibrosis, failed cell differentiation, and finally CKD. Conversely, CKD also plays an important role in the development of AKI. Both are associated with an increased risk of death and can result in serious kidney events such as end-stage renal disease.

Mechanisms of disease generation and progression in AKI and CKD remain incompletely understood (Singh et al., 2013; Tampe et al., 2017). Although several clinical studies have investigated early stage predictive biomarkers of kidney disease, few has been applied in clinical practice (Endre et al., 2013; Francoz et al., 2016; Peng et al., 2008; Soto et al., 2010). Our group identified urinary fractalkine as a marker of acute kidney transplant rejection (Peng et al., 2008). However, considerable challenges still lay ahead for the design and implementation of clinical kidney disease trials. Large sample size and long follow-up duration are essential in a multicenter clinical trial to guarantee the quality, efficiency, and safety of intervention and treatments as there are many different types and causes of kidney disease and treatment can be protracted (Luyckx et al., 2013). Moreover, serious complications greatly contribute to total mortality in kidney disease (Di Lullo et al., 2015; Pálsson & Patel, 2014; Ross & Banerjee, 2013), making it difficult to determine the major cause and best treatment. Thus, mature animal models are an indispensable part of scientifically designed kidney disease studies, and play an important role in resolving the bottleneck issue in treatment.

**Figure 2:**
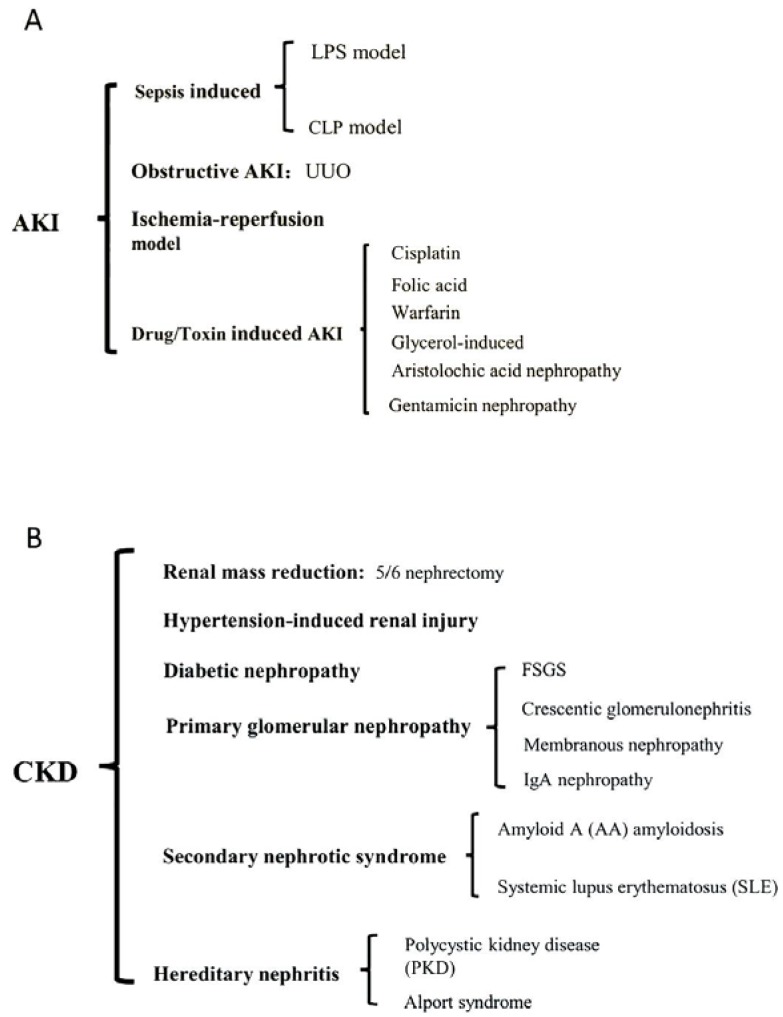
Summary of major acute kidney injury (AKI) and chronic kidney disease (CKD) models

Animal models have been extensively used to clarify the pathogenesis and underlying mechanisms of renal disease. Among these models, mice and rats are the most commonly used to study nephropathy events and potential therapeutic targets and to identify specific biomarkers of disease. Mice and rats are easily bred and are relatively inexpensive to house and maintain (Wei & Dong, 2012). Classic acute kidney disease can be induced in a variety of murine models by surgery or administration of drugs or toxins (Ortiz et al., 2015; Singh et al., 2012). Furthermore, genetically engineered mice and inbred strains provide a new platform for investigating complex human nephropathy (such as IgA nephropathy and diabetic nephropathy) (Marchant et al., 2015; Suzuki et al., 2014). In this review, we focused on murine models of AKI and CKD (Figure 2). 

A: Common causes of acute kidney injury (AKI). Traditional animal modeling approaches are included. B: Classification of chronic kidney disease (CKD). The following are available animal models for CKD; UUO: Unilateral ureteral obstruction; FSGS: Focal segmental glomerulosclerosis.

## ACUTE KIDNEY INJURY MODELS

Recently, several reviews of available models, including their advantages and disadvantages, have been discussed (Ortiz et al., 2015; Ramesh & Ranganathan, 2014); however, the types of models are incomplete and many details, such as model techniques and modeling time, are not mentioned. Current models of AKI can be induced by IR (pre-renal acute kidney failure), injection of drugs, toxins, or endogenous toxins (sepsis-associated AKI), and ureteral obstruction (post-renal acute kidney failure) (Sanz et al., 2013; Singh et al., 2012) (Table 1). This section will discuss experimental AKI models, surgical operations, model time courses, and drug/toxin dose ranges.

**Table 1:** Comparison of conventional acute kidney injury (AKI) mice models

Models	Species	Time-course/Dose range	Advantages	Disadvantages	References
Sepsis induced	Rats/mice	Cecal ligation and punctured to induce AKI; single i.p. dose of 10–15 mg/kg LPS are commonly used to induced AKI	Simple; inexpensive; standardized dose of LPS	Variable response between models; expected acute renal necrosis is not always achieved; AKI is not produced clinically and pathologically	Dejager et al., 2011; Liu et al., 2015; Liu et al., 2016
Ischemia-reperfusion	Rats/mice	Ischemia time: 30–45 min; reperfusion time: 24–48 h	High clinical relevance; classical model with high knowledge background	Surgery requires; reproducible outcome dependent on accurate Ischemia/ Reperfusion time	Hesketh et al., 2014; Wei & Dong, 2012
UUO	Rats/mice	1–2 weeks; longer time for renal fibrosis studies	Technically simple; reproducible	Surgery requires; not widely used as AKI mode; renal function can be compensated by the non-ligated kidney;	Bander et al., 1985; Chevalier et al., 2009; Ucero et al., 2014
Cisplatin	Rats/mice	Single does 6–20 mg/kg; cisplatin within 72 h to induce AKI	Simple and reproducible; similar to human renal disease	Requires higher does to induce AKI; the cisplatin use is decreased in clinical	Ko et al., 2014; Li et al., 2005; Morsy & Heeba, 2016; Xu et al., 2015
Aristolochic acid	Rats/mice	5 mg/kg/day Aristolochic acid for 5 days	Useful to study AKI-CKD transition;	No clinical correlate; less nephrotoxicity report	Matsui et al., 2011; Wu et al., 2014
Folic acid	Rats/mice	Single dose of 250 mg/kg induce AKI in 24-48 h	Simple and reproducible		Wen et al., 2012; Soofi et al., 2013
Warfarin	Rats	5/6 nephrectomy for 3 weeks and 8 days on warfarin	Clinically relevant; useful to study AKI caused by anticoagulants	Only modeled in rats	Brodsky, 2014; Ozcan et al., 2012; Ware et al., 2011
Glycerol	Rats/mice	Deprived of water for 24 h and single injection of 8–10 mg/kg 50% glycerol	Simple; reproducible	Severe pathology	Geng et al., 2014; Kim et al., 2014
Gentamicin	Rats	Dose range 40–200 mg/kg for 4–10 days	Highly relevant; reversible AKI	Requires higher dose of Gentamicin; different symptoms in human and rodents	Boroushaki et al., 2014; He et al., 2015; Heidarian et al., 2017

### Sepsis-associated AKI

Sepsis-associated AKI (SA-AKI) is characterized by severe inflammatory complications and high morbidity and mortality (Swaminathan et al., 2015). Frequently used experimental models of SA-AKI can be divided into two types: (1) injection of bacteria or endogenous toxins (e.g., LPS) into the peritoneum or blood; and (2) release of intestinal excreta by cecal ligation and puncture (CLP) or colon ascendens stent peritonitis (CASP) (Xu et al., 2014; Liu et al., 2015a).

**LPS models** LPS-induced AKI has mainly been studied in rats and mice. Compared with other species, rodents are significantly more resistant to the toxic or lethal effects of LPS. The dose of LPS commonly used in research is 10–15 mg/kg (Fink, 2014; Liu et al., 2015b; Venkatachalam & Weinberg, 2012). After LPS interacts with specific receptors such as Toll-like receptor 4 (TLR-4) (Solov'eva et al., 2013) on host immune cells, inflammatory cytokines like IL-1, TNF-α, and IL-6 are secreted, leading to hemodynamic alteration, widespread inflammation, and sepsis (Solov'eva et al., 2013). This is an acute model that usually terminates at 72–96 h.

**CLP model** The CLP model is the most frequently used model due to its simplicity. Firstly, ligation of the cecum from the distal to the ileocecal valve is made. After that, two needle punctures are made to extrude stool into the abdominal cavity (Liu et al., 2016b; Poli-De-Figueiredo et al., 2008). CLP in mice can develop the typical symptoms of bacterial peritonitis observed in humans and yield good results (Fink, 2014). However, it is difficult to control the severity of sepsis and the differences in age and strain in CLP models (Zarjou & Agarwal, 2011). Moreover, reproducible AKI cannot be developed in a CLP model (Dejager et al., 2011). 

Although experimental models have extended our understanding of sepsis and sepsis-associated AKI, there is still no effective clinical therapy (Alobaidi et al., 2015). Several clinical trials targeting specific signaling pathways based on convincing results in murine models have failed to improve survival in septic patients. 

### Ischemia-reperfusion (IR) model

Currently, IR is the most widely used model for clinical AKI and renal transplant studies (Hesketh et al., 2014). Among the variety of existing models, the mouse clamping model is often applied due to its low costs and choice of transgenic models (Sanz et al., 2013). According to previous studies, commonly used models contain bilateral renal IR (Huang et al., 2012; Hu et al., 2010; Kim et al., 2012) and unilateral renal IR (Braun et al., 2012; Chen et al., 2011; Gall et al., 2011).

First, 50–60 mg/kg of pentobarbital (5 mg/mL) is used to anesthetize mice by intraperitoneal (i.p.) injection, with body temperatures then maintained at 36.5–37 °C during surgery. Second, the renal artery and vein are clamped by micro-aneurysm clips for a variable length of time to induce different severities of kidney injury. In general, clamping the renal pedicle for 30 min is used to induce IR injury (Huang et al., 2015; Liu et al., 2016a). Successful ischemia can be confirmed by gradual darkening of the kidney (from red to dark purple). The clamp is then removed at the desired time to achieve reperfusion, with the kidney color immediately reverting to red (Hesketh et al., 2014; Wei & Dong, 2012). Ischemia-reperfusion will trigger tubular cell necrosis and apoptosis, inflammation, and oxidative stress (Rovcanin et al., 2016; Sanz et al., 2013; Zhou et al., 2015), which can result in a decline of renal function, as evaluated by blood urea nitrogen (BUN) and serum creatinine. Despite the view that the IR model is less stable, experimental factors such as anesthesia dose, mouse strain, age, gender, and feeding conditions can be well-controlled (Wei & Dong, 2012).

### Obstructive AKI

Unilateral ureteric obstruction (UUO) is the most common rodent model used to study AKI and CKD (Ucero et al., 2014). This model can result in hydronephrosis and blood flow changes. Ischemia, hypoxia, and oxidative stress (Dendooven et al., 2011) contribute to the tubular cell death, followed by interstitial inflammation. Additionally, transformed fibroblasts can interact with extracellular matrix deposition to cause renal fibrosis (Xiao et al., 2016; Zhou et al., 2014).

Recent studies using the UUO model have shown that adenosine levels (Tang et al., 2015), nuclear factor-erythroid-2-related factor 2 (Nrf2) (Chung et al., 2014), interleukin-10 (Jin et al., 2013), and the JAK/STAT signaling pathway (Koike et al., 2014) are related to renal fibrosis, thus offering a potential therapeutic target for renal injury. The UUO model is relatively straightforward. Male animals, which are recommended in this model, undergo a midline abdominal incision under anesthesia, with the left ureter then ligated with 4–0 silk. After 24 h, the ureter obstruction is removed (Bander et al., 1985). Different from the complete UUO model, a partial UUO is created by inserting the ureter into a surgically created tunnel in the psoas muscle (Sugandhi et al., 2014). Reversible partial UUO is generally performed in neonatal mice to investigate kidney recovery after obstruction (Ucero et al., 2014). However, the complete UUO model is more popular because it is less technical and more easily reproduced.

### Toxin-induced AKI

Exogenous drugs or poisons and endogenous toxins are used to stimulate AKI by their side or poisoning effects. Among these models, 6–20 mg/kg cisplatin can result in acute tubular injury within 72 h, whereas administration of 40–200 mg/kg gentamicin in rats for 4–10 d can induce acute renal failure. Aristolochic acid and high dose folic acid (FA) are frequently used to study AKI-CKD transition, with AKI models developed by warfarin and glycerol also used.

### Cisplatin-induced AKI

Cisplatin is a chemotherapy agent that is widely used in the treatment of solid tumors (Karasawa & Steyger, 2015). However, high doses of cisplatin can induce prominent nephrotoxicity in humans (Humanes et al., 2012; Malik et al., 2015). Among cisplatin’s adverse effects, direct proximal tubular toxicity is significant. Tubular cell necrosis and apoptosis are mediated by inflammation, oxidative stress, and calcium overload. These modes of cell death both lead to increased vascular resistance and decreased GFR (Ozkok & Edelstein, 2014). The pathology and recovery phase of cisplatin-induced AKI models are comparable with those of humans. Many studies have reported that single i.p. injection of 6–20 mg/kg cisplatin can induce AKI within 72 h in rodent models (Ko et al., 2014; Lee et al., 2009; Morsy & Heeba, 2016; Xu et al., 2015). Furthermore, other groups have developed AKI models by injecting higher doses of cisplatin, including 30 mg/kg, i.p. (Lu et al., 2008; Mitazaki et al., 2009) and 40 mg/kg, i.p. (Zhang et al., 2016). Based on results from this experimental model, several therapeutic targets have been established.

### Aristolochic acid nephropathy

It has been reported that i.p. injection of aristolochic acid (AA) (5 mg/kg/d for 5 d) can induce AKI (Matsui et al., 2011; Wu et al., 2014). The pathology of acute aristolochic acid nephropathy (AAN) involves proximal tubular cell injury and necrosis with oxidative stress and progressive interstitial renal fibrosis (Baudoux et al., 2012; Nortier et al., 2015; Yang et al., 2010). Rabbit and rat models were first used to recapitulate human CKD and confirmed that aristolochic acid is related to Chinese herb nephropathy and Balkan endemic nephropathy (De Broe, 2012; Sanz et al., 2013). Recently, studies on AA in AKI-CKD transition have increased. Signaling pathways such as nuclear factor erythroid 2-related factor 2 (Nrf2) (Wu et al., 2014) and Jun N-terminal kinases (JNK) signaling (Rui et al., 2012; Yang et al., 2010) have been shown to play important roles in AA-induced acute kidney lesions, thus providing several new therapeutic targets.

### Folic acid-induced AKI 

A high dose of FA can also induce AKI in mice (Wen et al., 2012). Intraperitoneal injection of 250 mg/kg of FA (dissolved in 0.3 mmol/L NaHCO_3_) can cause acute renal toxicity and injury in rodents (Soofi et al., 2013; Wen et al., 2012). The mechanism of FA nephropathy might be due to FA crystal deposition in the tubular lumen, which results in obstruction and extensive necrosis (Kumar et al., 2015; Szczypka et al., 2005). A more recent study showed that inhibition of ferroptosis can protect kidneys from FA-induced AKI, implicating its important role in FA nephropathy (Martin-Sanchez et al., 2017). Additionally, mitochondrial dysfunction and early renal fibrosis, which are related to CKD pathology, can be found in the FA-induced AKI model, thus providing a new way in which to investigate AKI-CKD transition (Stallons et al., 2014).

### Warfarin-induced AKI

A new model of warfarin-induced hematuric AKI based on 5/6 renal nephrectomized rats (Ware et al., 2011) was established to study the pathology of warfarin-related nephropathy (WRN) in patients with excessive anticoagulant (Rizk & Warnock, 2011). The 5/6 nephrectomy was performed in Sprague Dawley rats, with animals allowed three weeks recovery from the surgery before warfarin treatment. Warfarin was given orally via drinking water, and warfarin dosage was based on rat weight (Brodsky, 2014). Extensive glomerular hemorrhage and tubular obstruction can occur in rats after seven days administration of warfarin (0.4 mg/kg/d), as well as increased serum creatinine (Brodsky, 2014; Ozcan et al., 2012). Besides, WRN can also induce AKI, accelerate CKD, and increase the mortality rate in warfarin-treated patients (Brodsky et al., 2011). However, the mechanism and therapeutic strategies to ameliorate WRN-induced AKI remain to be demonstrated.

### Glycerol-induced AKI

Rhabdomyolysis is a syndrome in which the breakdown of skeletal muscle leads to the release of intracellular proteins and toxic compounds into circulation (Hamel et al., 2015). AKI is a common complication of rhabdomyolysis and accounts for the high mortality (Elterman et al., 2015; Zhang et al., 2012). Presently, oxidative damage and inflammation are the two major causes of rhabdomyolysis-induced AKI (Tomino, 2014). To reproduce the typical symptoms observed in humans, rats or mice are deprived of water for 24 h, after which a 8–10 mL/kg dose of 50% glycerol is administrated in the hindlimb muscle (Geng et al., 2014; Kim et al., 2014b). Although studies have reported that vitamin C (Ustundag et al., 2008), L-carnitine (Aydogdu et al., 2006; Ustundag et al., 2009), and resveratrol (Aydogdu et al., 2006) can ameliorate rhabdomyolysis-induced AKI, there is currently no effective therapy for this disease except aggressive rehydration (Gu et al., 2014).

### Gentamicin nephropathy 

Gentamicin is an aminoglycoside antibiotic commonly used to prevent gram-negative bacterial infection. Nevertheless, nephrotoxicity limits its use in clinical practice (He et al., 2015). Doses of gentamicin ranging from 40–200 mg/kg administered for 4–10 d (Bledsoe et al., 2008; Boroushaki et al., 2014; Heidarian et al., 2017; Jabbari et al., 2011) can induce acute renal failure in rats. Administration of 100 mg/kg i.p. for 5 d is recommended to mimic gentamicin-induced nephrotoxicity (Hur et al., 2013; Stojiljkovic et al., 2008; Stojiljkovic et al., 2012). This acute model is characterized by increased levels of serum urea and creatinine, decreased GFR, tubular lesions, and fibrosis (Romero et al., 2009; Al-Shabanah et al., 2010; Balakumar et al., 2010).

## CHRONIC KIDNEY DISEASE MODELS

CKD models mainly include diabetic/hypertensive nephropathy, glomerular injury, polycystic kidney disease (PKD), and chronic tubulointerstitial nephritis (Table 2). In this section, key information on various rodent models of CKD is discussed.

**Table 2:** Advantages and disadvantages of experimental CKD mice models

Pathology	Models	Advantages	Disadvantages	Reference
Renal mass reduction	5/6 nephrectomy (rats)	Mimics the progressive renal failure; after loss of renal mass in human	Highly influenced by back ground strains; surgery requires	Ergur et al., 2015; He et al., 2012; Kim et al., 2009
Hypertension	SHR rats+UNX; angiotensin II infusion models	Highly relevant to hypertension nephropathy; useful to study AngII effect over kidney	Surgery requires; high cost; slow progression	Guo et al., 2015; Lankhorst et al., 2015; Zhong et al., 2016
Diabetic nephropathy	Streptozotocin mice/rats; NOD mice BB-DP rat; ob/ob mice db/db mice; DBA/2J mice; STZ-eNOS^-/-^; db/db-eNOS/ mice	Gene modified; commercially available; available on multiple strains	No ideal model to mimics; diabetic nephropathy; expensive; some strains are infertile	Betz & Conway, 2014; Graham & Schuurman, 2015; Kitada et al., 2016; Ostergaard et al., 2017
Primary glomerular nephropathy; focal segmental glomerulosclerosis	Adriamycin (rat, mice) models; Puromycin (rat) models	Widely used; induce podocyte injury	Highly depends on species and strains; toxic for most other cells	De Mik et al., 2013; Hakroush et al., 2014; Lee & Harris, 2011; Wada et al., 2016
Crescentic glomerulonephritis	Nephrotoxic nephritis model; anti-GBM nephritis model	Similar to human Crescentic glomerulonephritis	Single symptom; difficult to induce	Borza & Hudson, 2002; Cheungpasitporn et al., 2016
Membranous nephropathy	heymann nephritis rats; Cationic BSA mouse model	Widely used; identical pathology; marked proteinuria	Antigen (megalin) not found in human MN; limited experience	Cybulsky, 2011; Jefferson et al., 2010; Motiram Kakalij et al., 2016
IgA nephropathy	ddY mouse, HIGA mice Uteroglobin-deficient mice CD89-transgenic mouse	Reproduces human pathology; multiple models available	Mild disease development usually without progression towards end-stage renal disease	Eitner et al., 2010; Papista et al., 2015; Suzuki et al., 2014
Secondary nephrotic syndrome; Amyloid A (AA) amyloidosis	Injection of chemical or biological compounds models	Widely used; reproduce features of human diseases	Rarely develop renal failure	Kisilevsky & Young, 1994; Teng et al., 2014
Systemic lupus erythematosus	NZB/NZW F1 mice MRL and CD95 mutants model	Widely used; marked proteinuria	Incomplete features of SLE	Fagone et al., 2014; Nickerson et al., 2013; Otani et al., 2015
Hereditary nephritis; polycystic kidney disease; Alport syndrome	*pkd1* or *pkd2* gene engineered mouse; *COL4A4*3 gene knockout mouse	Widely used and useful to study PKD; major model; develop proteinuria and renal failure		Kashtan & Segal, 2011; Ko & Park, 2013; Korstanje et al., 2014; Ryu et al., 2012

### Renal mass reduction

The remnant kidney model has been one of most commonly used experimental models of CKD. The 5/6 subtotal nephrectomy approach is widely used to mimic human CKD in rats. The right kidney is removed and the upper and lower poles (2/3 of the left kidney) are resected after ligation of the left renal artery (He et al., 2012). After surgery, activation of the renin-angiotensin system (RAS) can cause glomerular hypertension/hyperfiltration (Ergür et al., 2015; Tapia et al., 2012). Together with oxidative stress and inflammation, the glomerular hypertension/hyperfiltration finally results in glomerulosclerosis, tubulointerstitial injury, renal atrophy, proteinuria, and possible ESRD (Gong et al., 2016; Kim et al., 2009). The remnant kidney model is highly influenced by the animal strain used. C57BL/6 mice are resistant to fibrosis or progressive CKD, whereas other animal strains such as rats and CD-1, 129/Sv, and Swiss-Webster mice are susceptible (Leelahavanichkul et al., 2010; Orlando et al., 2011). In addition, high mortality and little renal tissue after 5/6 nephrectomy are also challenges to this model.

### Diabetic nephropathy 

Diabetic nephropathy (DN) is the leading cause of ESRD. There are many kinds of rodent models relevant to diabetic nephropathy, but none of them perfectly mimics the human disease (Deb et al., 2010). The Animal Models of Diabetic Complications Consortium (AMDCC) defines the ideal rodent model of human diabetic nephropathy and complications (Kong et al., 2013; Kitada et al., 2016). The latest validated criteria are: (1) more than 50% decrease in GFR; (2) greater than 10-fold increase in albuminuria compared with controls; and (3) pathological changes in kidneys including advanced mesangial matrix expansion±nodular sclerosis and mesangiolysis, glomerular basement membrane (GBM) thickening by >50% over baseline, arteriolar hyalinosis, and tubulointerstitial fibrosis. Classical type 1 diabetes can be modeled by the administration of streptozotocin (a toxin to β-cells that results in insulin deficiency), with spontaneous autoimmunity (e.g., NOD mice or BB-DP rat) or with gene mutation (Akita and OVE26 mice) (Graham & Schuurman, 2015; Kitada et al., 2016). A high fat diet is commonly used to induce obesity and insulin resistance and develop glomerular lesions in mice (Soler et al., 2012). Typical type 2 diabetes nephropathy (DN) model can be establised by leptin deficiency (e.g., ob/ob mice) or inactivation of the leptin receptor (e.g., db/db mice, Zuker rat) (Soler et al., 2012). To exhibit more pathological features of human DN, recent studies have focused on (1) targeted gene knockout in mice (e.g., eNOS-deficient mice (Takahashi & Harris, 2014)), (2) selection of more susceptible rodent species and strains (e.g., FVB (Chua et al., 2010) and DBA/2J mice (Østergaard et al., 2017), and (3) monogenic manipulations or superimposing additional key factors to accelerate nephropathy (e.g., STZ-eNOS^-/-^, db/db eNOS^-/-^) (Betz & Conway, 2014; Nakayama et al., 2009).

### Hypertension-induced renal injury

Spontaneously hypertensive rats are usually used to investigate hypertension-induced nephropathy. Additionally, unilateral nephrectomy is required to promote significant renal injury with increased glomerular pressure and flow (Zhong et al., 2016). Chronic injection of angiotensin II for weeks also results in persistent hypertension and renal injury (Dikalov et al., 2014). Vascular endothelial growth factor (Lankhorst et al., 2015), Smad signaling (Liu & Davidson, 2012a), and inflammatory cytokines (Guo et al., 2015) are also involved in this process.

### Primary glomerular nephropathy

#### Focal segmental glomerulosclerosis (FSGS)

FSGS is a common primary glomerular disorder characterized by podocyte injury and loss and marked proteinuria (Fogo, 2015). Although there is currently no primary FSGS model available, several secondary FSGS models have been established. Adriamycin (ADR) and puromycin are widely used to study FSGS. Single injection of these specific toxins can result in podocyte foot process effacement, deficient filtration barrier, and nephrotic syndrome (Fogo, 2003; Zhang et al., 2013). However, the dosage of adriamycin is highly dependent on species and strain. Most rat species are susceptible to low doses of ADR ranging from 1.5–7.5 mg/kg (Lee & Harris, 2011), whereas most mouse strains are resistant to ADR. To produce a successful model, higher doses of ADR are required, for example 9.8–12 mg/kg in male BALB/C (Wada et al., 2016) and 13–25 mg/kg in C57BL/c mice (Cao et al., 2010; Hakroush et al., 2014; Jeansson et al., 2009; Maimaitiyiming et al., 2016; Wang et al., 2000).

Gene modification approaches in mice, such as inactivation of Mpv-17 (Casalena et al., 2014; Viscomi et al., 2009), knockout α-actinin-4 (De Mik et al., 2013; Henderson et al., 2008) or NPHS2 (Mollet et al., 2009), or introducing the expression of Thy-1.1 antigen on podocytes, can also lead to proteinuria and FSGS (Smeets et al., 2004).

### Crescentic glomerulonephritis

Antibodies fixation in the whole glomeruli (nephrotoxic nephritis) or GBM (anti-GBM nephritis) are the primary models used to mimic human crescentic glomerulonephritis (Hénique et al., 2014). Intraperitoneal injection of heterologous antibodies to heterologous whole glomeruli can induce nephrotoxic nephritis (Gigante et al., 2011). Anti-GBM nephritis can be caused by immunization with the non-collagenous domains of the alpha-3 chain of type IV collagen or passive transfer of anti-GBM antibodies (Cheungpasitporn et al., 2016; Kambham, 2012; Kvirkvelia et al., 2015). After treatment, severe proteinuria and azotemia appear in the following weeks.

### Membranous nephropathy

Membranous nephropathy (MN) is a major cause of nephrotic syndrome in the elderly and is characterized by subepithelial deposits and diffuse thickening of the GBM (Makker & Tramontano, 2011). Active and passive Heymann nephritis model in rats closely resemble human MN and have been used to study MN (Sendeyo et al, 2013).

Autologous antibodies are exposed to target antigens by injection of kidney extracts or antiserum to antigen generated in another animal species (Cybulsky, 2011; Jefferson et al., 2010), resulting in immune deposits associated with heavy proteinuria (Cybulsky et al., 2005). In rat models, megalin and receptor associated protein (RAP) are the major podocyte antigens targeted by the circulating antibodies (Ronco & Debiec, 2010). However, studies have shown that megalin is neither expressed in human podocytes nor detected in patients with membranous nephropathy (Beck & Salant, 2010; Ma et al., 2013). Recently, M-type phospholipase A2 receptor (PLA2R) was identified as a target antigen for autoantibodies in human MN (Debiec & Ronco, 2011; Herrmann et al., 2012; Kao et al., 2015). Additionally, circulating thrombospondin type-1 domain-containing 7A (THSD7A) has been detected in a subgroup of patients with idiopathic MN rather than PLA2R, suggesting a new target antigen in human MN (Tomas et al., 2014).

The cationic BSA mouse model also produces features of human MN. Mice are preimmunized with cationic bovine serum albumin (cBSA) every other day for a week. Two weeks later, mice are reimmunized with cBSA in Freund’s adjuvant (Motiram Kakalij et al., 2016). Mice will develop symptoms of MN, including severe proteinuria, diffuse thickening of the GBM, subepithelial deposits, and GBM spikes.

### IgA nephropathy (IgAN)

IgAN is the most common form of glomerulonephritis, and is characterized by mesangial immune complex depositions that contain IgA1, IgG, complement C3, and IgM (Daha & Van Kooten, 2016). Inducible IgAN models include intravenous injection of IgA containing immune complexes to develop mild and transient IgAN (Rifai et al., 1979), and oral administration of protein antigens that result in mesangial IgA deposits (Emancipator et al., 1983). The ddY mouse is a spontaneous IgAN model derived from a non-inbred strain that develops glomerulonephritis and mild proteinuria without hematuria (Suzuki et al., 2014). Mouse line HIGA, an inbred strain with high levels of circulating IgA, shows significant early-onset immune deposits (Eitner et al., 2010). Other genetically modified mice, such as uteroglobin-deficient mice (Lee et al., 2006) and CD89-transgenic mice (Moura et al., 2008; Papista et al., 2015), can also be used to investigate IgA nephropathy. Although some models are available, the underlying mechanism of IgAN is still not fully understood.

### Secondary nephrotic syndrome

In this section, murine models of systemic lupus erythematosus and amyloidosis are reviewed. Transgenic murine models are widely used to investigate these complex diseases, especially systemic lupus erythematosus (SLE). Both MRL and CD95 gene mutant animals can serve as research models to develop SLE symptoms and investigate potential therapies.

### Amyloid A (AA) amyloidosis

Amyloid A (AA) amyloidosis is a serious complication of chronic inflammation. AA-type amyloid deposition can cause alteration in tissue structure and function, with the kidney noted to be a major target organ (Simons et al., 2013). Injection of chemical or biological compounds such as casein, lipopolysaccharide (Kisilevsky & Young, 1994; Skinner et al., 1977), an extract of amyloidotic tissue or purified amyloidogenic light chains (Teng et al., 2014) are widely used to create AA amyloidosis mouse models. However, unlike clinical AA-amyloid patients, these models rarely develop renal failure (Simons et al., 2013). In recent years, a striking transgenic murine model has been developed. Mice carrying the human interleukin-6 gene under the control of the metallothionein-I promoter or with doxycycline-inducible transgenic expression of SAA provide another way to investigate AA-amyloid (Simons et al., 2013).

### Systemic lupus erythematosus (SLE)

Lupus nephritis is characterized by autoantibodies against nuclear autoantigens such as DNA, histones, and nucleosomes (Liu & Davidson, 2012b). Most studies on SLE are based on murine models. Genetically modified models include MRL and CD95 mutants such as MRL^lpr^ and FasL^gld^ mice (Nickerson et al., 2013; Otani et al., 2015), BXSB mice (McGaha et al., 2005; McPhee et al., 2013), and NZB/NZW F1 mice (Fagone et al., 2014), which are widely used to develop proteinuria, lymphoproliferation, and similar features relevant to human lupus nephritis (McGaha & Madaio, 2014). Recently, TWEAK-Fn14 signaling has been reported to play an important role in the progression of lupus nephritis and anti-TWEAK blocking antibodies can preserve renal function and increase survival rate in experimental models of CKD (Gomez et al., 2016; Sanz et al., 2014). Although multiple mouse models have been used to investigate lupus nephritis, each model has limitations that impede our understanding of the pathogenesis and clinical manifestations of this disease. Subsequently, no effective therapy for lupus nephritis currently exists.

### Hereditary nephritis

#### Polycystic kidney disease (PKD) 

PKD includes a group of human monogenic disorders inherited in an autosomal dominant (ADPKD) or recessive (ARPKD) fashion. PKD is mainly restricted to the liver and kidney, and occurs in a range of ages from children to the elderly. In children and adults, ADPKD and ARPKD are the most common genetic nephropathies and leading causes of ESRD (Liebau & Serra, 2013). ADPKD is caused by mutation of either PKD1 (85%) or PKD2 (15%) (Kim et al., 2014a), whereas ARPKD is caused by PKHD1 gene mutations (Sweeney & Avner, 2011). Although hereditary PKD is complex and diverse, it is normally induced by single mutations in single genes. Therefore, genetically engineered murine models are widely used to mimic human PKD. As homozygous mice of PKD1 or PKD2 result in embryonic lethality (Woudenberg-Vrenken et al., 2009), conditional knockouts, inducible strategies, or the introduction of unstable alleles are the major ways to establish experimental models (Ko & Park, 2013). There have been some successful clinical trials based on results from these models. For example, tolvaptan has been proven to be effective in ADPKD and is now marketed in Japan (Torres et al., 2012). Moreover, combination therapy of tolvaptan and pasireotide has brought significant reduction in cystic and fibrotic volume in a PKD1 mouse model (Hopp et al., 2015).

#### Alport syndrome

Alport syndrome (AS) is a hereditary glomerulopathy resulting from mutations in the type IV collagen genes *COL4A3*, *COL4A4*, and *COL4A5*, and is characterized by hematuria, renal failure, hearing loss, ocular lesions (Savige et al., 2011), and abnormal collagen IV composition in the GBM (Savige et al., 2013). The *COL4A4*3 gene knockout mouse is the major model used to study the pathogenesis of AS. Homozygous mice can develop proteinuria at 2–3 months of age and die from renal failure at 3–4 months (Kashtan & Segal, 2011). In *COL4A4*3^-/-^ mice, studies have shown that TNF-α contributes to Alport glomerulosclerosis by inducing podocyte apoptosis (Ryu et al., 2012). Furthermore, spontaneous *COL4A4* mutation in NONcNZO recombinant inbred mice exhibits early stage proteinuria associated with glomerulosclerosis. These genetically modified mice provide valuable models for potential therapy testing and help understand the mechanisms of AS (Korstanje et al., 2014).

## CONCLUSIONS

Although AKI and CKD are significantly increasing worldwide and cause high mortality, clinical diagnosis and therapeutic interventions are lagging. AKI-CKD transition and the underlying mechanisms of complex CKD such as IgA nephropathy, diabetic nephropathy, and FSGS are still unclear and impede the search for potential therapies. Despite the valuable new insights into kidney disease gained from existing models, many do not fully reproduce human clinical diseases. Thus, improved murine models are still desperately needed to investigate potential diagnostic and therapeutic approaches. In AKI models, obtaining new mouse strains susceptible to toxins/drugs is urgent, and finding new approaches to develop stable and reproducible AKI models is necessary. As for CKD models, to develop complex and specific pathologies, mice with multiple genetically modified will be widely used to develop complex and specific pathology in the near future. Additionally, models that faithfully develop common conditions such as DN or SLE are also imperative.
